# Association of husbands' education status with unintended pregnancy in their wives in southern Ethiopia: A cross-sectional study

**DOI:** 10.1371/journal.pone.0235675

**Published:** 2020-07-09

**Authors:** Canaan Negash Seifu, Paul Patrick Fahey, Tewodros Getachew Hailemariam, Evan Atlantis

**Affiliations:** 1 School of Public Health, Wolaita Sodo University, Wolaita Sodo, Ethiopia; 2 School of Nursing and Midwifery, Western Sydney University, Sydney, New South Wales, Australia; 3 School of Health Science, Western Sydney University, Sydney, New South Wales, Australia; 4 Translational Health Research Institute, Western Sydney University, Sydney, New South Wales, Australia; 5 School of Medicine, University of Adelaide, Adelaide, South Australia, Australia; Graduate School of Public Health and Health Policy, City University of New York, UNITED STATES

## Abstract

**Background:**

Unintended pregnancy rates are substantially higher in developing regions, have significant health consequences, and disproportionately affect subgroups with socio-economic disadvantage. We aimed to examine whether there is an association between husbands’ education status and their wives unintended pregnancy in southern Ethiopia.

**Methods:**

The data source for this study was from a cross-sectional study on iron-folate supplementation and compliance in Wolaita, South Ethiopia. Data were collected from October to November 2015 in 627 married pregnant women regarding their husbands’ education status, socio-demographic characteristics, and if they wanted to become pregnant at the time of survey using an interviewer administered questionnaire. Logistic regression was used to estimate Odds Ratios (ORs) with associated z-tests and 95% Confidence Intervals (95% CI) for variables associated with unintended pregnancy.

**Results:**

The proportion of unintended pregnancy in this sample was 20.6%. Husbands’ education status, age, residence, and using family planning methods were associated with unintended pregnancy (all P-values < 0.05). Multivariable models consistently showed that being married to a husband with at least some college or university education was associated with a decreased OR for unintended pregnancy after controlling for age and use of family planning at conception period (OR 0.36 [95%CI: 0.17, 0.82]) and age and rural residence (OR 0.40 [95%CI: 0.18, 0.90]).

**Conclusion:**

Unintended pregnancy among Ethiopian woman was consistently associated with being married to least educated husbands in southern Ethiopia. Increasing age and living in a rural vs urban area were also independently associated with unintended pregnancy. Strategies for addressing family planning needs of women with poorly educated husbands should be the subject of future research.

## Introduction

Unintended pregnancies are associated with an increased health and economic burden, especially in developing regions [1]. An unintended pregnancy is typically defined as a pregnancy that is either unwanted (the pregnancy was not desired) or mistimed (the pregnancy was earlier than desired). The incorrect use or non-use of effective contraceptives is a major risk factor for unintended pregnancy [2, 3]. It is estimated that 44% of all pregnancies worldwide were unintended for the survey period 2010–14, of which East Africa had the second highest rate [1].

There are several health risks associated with unintended pregnancy for both the mother and the newborn. These include, but are not limited to, abortion, pre-eclampsia, postpartum haemorrhage, maternal death, and preterm birth [4]. Unintended pregnancies that occur in developing countries are associated with an increased risk of unsafe abortions, which is exacerbated by poor access to appropriate health care [1]. Indeed, almost all (97%) unsafe abortions occur in developing countries, of which Africa has the highest risk of related deaths [5]. In Ethiopia, a national reproductive health strategy which aims to increase access to and use of family planning services commenced in 2006 [6]. Despite this, 22% of married women reportedly had unmet needs for family planning and 17% of pregnancies were mistimed while 8% were unwanted in 2016 [7]. Approximately 25% of all female deaths due to pregnancy-related causes [7] might have been preventable if more effective family planning services were widely available and used. Further research of high-risk subgroups and predictors of unintended pregnancy is needed to inform family planning services and policies in both Ethiopia and developing counties in general.

Socioeconomic disadvantage, including limited education, may be an important risk factor for unintended pregnancies [8]. This may be particularly relevant in the typical rural Ethiopian family where the husband is the dominant decision maker on most aspects of life [9, 10]. Dominant decision-making by the husband could impact the reproductive life of women [11]. For instance, one study using demographic and health surveys of 27 Sub-Sahara African countries, including Ethiopia, revealed that women were less likely to make reproductive health related decisions by themselves if married to a husband with no formal education [12]. Another study in Ethiopia showed that women tended to be involved in their own health care decisions when their husbands had completed more years of education [10].

While increasing age, lower maternal education, having no paid job, respondent’s religion, and family size have all been shown to be associated with unintended pregnancy [13–21], the relationship between the husbands’ education status and the occurrence of unintended pregnancy in Ethiopia is still unclear. Therefore, we aimed to examine the association between husbands’ education status and unintended pregnancy events among their wives and whether or not information on husbands’ education status was sufficient to identify high risk groups in South Ethiopia. We hypothesised that lower education status in husbands would be associated with higher rates of unintended pregnancies in their wives.

## Materials and methods

We present this paper according to guidance from the STROBE Statement for reporting cross-sectional studies [22] and the Journal’s formatting requirements.

### Study design

We used a cross-sectional study design that was part of a survey conducted to assess the adherence to recommended iron-folic acid intake and its predictors among pregnant women attending antenatal care (ANC) in Wolaita, southern Ethiopia [23].

### Study setting and participants

The study was conducted from October to November 2015 in eight health centers that are located in three Woredas (districts) and two administrative towns in Wolaita Zone, southern Ethiopia. Wolaita is located 327 km south of Addis Ababa–the capital city. All pregnant women who were 15 years and above that visited the health centers were potentially eligible for this study. There were no exclusion criteria other than not providing consent. The number of pregnant women to recruit from each health center was allocated proportionally to the number of pregnant women who attended in the month prior to data collection. The study participants were recruited after their ANC consultation using a systematic random sampling procedure. The data collector approached every second pregnant woman at the completion of their ANC service, provided them with information about the study purpose and invited their participation. A total of 662 pregnant women were invited to participate in the survey of which 647 (97.7%) provided full or partial responses. In the current study, only married pregnant women who had complete data (n = 627) were selected for analysis.

### Variables

#### Outcome

Unintended pregnancy is defined as a negative answer to the question “Do you want to be pregnant?”. Response options were ‘yes’ or ‘no’.

#### Predictors

Participants were asked to select the best description of their husbands’ education status from the categories (no formal education, at least some primary, at least some secondary or at least some college or university).

Socio-demographic characteristics of the respondent recorded were age in years, religion (Protestant, Orthodox, Catholic and Muslim), educational status (no formal education, at least some primary, at least some secondary or at least some college or university), residence (urban/rural), income sources (respondent has her own source of income: yes/no), and main source of household income (farming/working for a wage/self-employed excluding farming).

Reproductive health-related characteristics were ever use of family planning (yes/no) and family planning use at the time of conception (yes/no).

#### Data collection methods

A structured, interviewer-administered and pretested questionnaire was used to collect all data and is available in both English and Amharic language (S1 Appendix). The questionnaire was pre-tested on 34 pregnant women in non-sampled health institutions in the same study area. Based on the result of pre-test, minor changes (such as using simple instead of technical words) were made on the questionnaire. Prior to data collection, five undergraduate nursing students who had experience in data collection were trained for one day on how to administer the questionnaire. The data collectors used the final questionnaire to administer the survey in a place or room within the health institution that provided maximum privacy to participants.

#### Statistical analysis

We describe our sample of pregnant women in southern Ethiopia using frequency counts and percentages for categorical variables and mean and standard deviation for age. The small number of data records that had missing values were excluded.

The outcome variable, unintended pregnancy yes/no, is dichotomous and all analyses were conducted using logistic regression. As the primary aim was to test husbands’ education status as a predictor of unintended pregnancy, it was included in all of the analyses. The secondary aim was to develop the best possible prediction model which included husbands’ education status. Our criteria for best model was lowest deviance (-2 log likelihood) statistic while retaining husband’s education as a predictor.

Our preliminary analyses indicated that there were strong associations between predictor variables; for example, educational status of participants was associated with residence (X^2^ = 179.2, df = 3, P < 0.001) and having own source of income (X^2^ = 53.16, df = 3, P < 0.001) while, husband’s education status was associated with residence (X^2^ = 180.8, df = 3, P < 0.001) and family size (X^2^ = 139.9, df = 3, P < 0.001).

Given this, starting with husband’s education status as a predictor of unintended pregnancy and we then systematically tested each other potential predictor for ability to improve the model. That is, we added each separately to the logistic model and tested for improvement in fit with a likelihood ratio test (the decrease in the deviation between the predictions from the model and the observed outcome) without obscuring the effect of husband’s education. The next most important predictor to add to the model was selected considering both statistical significance and clinical importance of the predictor [24]. The process was repeated to build more complex models.

We gave preference to larger categories when choosing the reference categories for the odds ratios (ORs). Associations with predictor variables are presented as ORs, with associated 95% confidence intervals (95%CI), p-values, and likelihood ratio statistics with associated degrees of freedom and p-values. All data were entered into Epi Info (TM) 3.5.4 (Centers for Disease Control and Prevention) and exported to SPSS version 21 (IBM® SPSS® Statistics, IBM Corp, New York) for statistical analysis.

#### Sample size estimation

As the data were collected for a different purpose (iron-folic acid) there is no *a priori* sample size calculation for unintended pregnancies. Assuming an unintended pregnancy rate of 20%, the study had 80% power to detect a 12% difference in prevalence of unintended pregnancy.

*Ethics approval and consent to participate*. The study protocol was approved by Wolaita Sodo University, College of Health Sciences Ethical review committee. As approved by the Ethics committee, informed consent was obtained orally before the interview since the study area was rural and the literacy was low. The committee was informed about the study area and had approved the use of oral consent. Before the study started, the purpose of the study was explained for study participants and only those who gave their consent were able to be part of the study. Moreover, an informed consent form was signed by the data collector to document that oral consent was given by the participant. Name of study participants was not collected. Dual enrolment is unlikely as it is very unlikely in Ethiopia for the pregnant women to have more than one ANC visit in the study timeframe. Also, each interview commenced with a clear statement of the name of the study and women were questioned as to whether they had already participated.

## Result

A total of 627 pregnant married women were included in the analysis. Characteristics of the study participants are summarised in the table (Table 1). Overall, 20.6% (129/627) women reported that their current pregnancy was unintended.

**Table 1:** Characteristics of the study sample and unadjusted odds ratios for unintended pregnancy.

Variables		Total	Unintended pregnancy		Unadjusted OR (95% CI)
		N = 627	No (n = 498)	Yes (n = 129)	
Age, mean (SD)		26.9 (5.5)	26.1 (5.3)	29.9 (5.2)	1.14 (1.09, 1.19) ***
Educational status, n (%)	No Formal Education	262 (41.8)	181 (36.3)	81 (62.8)	1
	Primary	157 (25.0)	133 (26.7)	24 (18.6)	0.4 (0.2, 0.67) ***
	Secondary	139 (22.2)	122 (24.5)	17 (13.2)	0.31 (0.18, 0.55) ***
	College or university	69 (11.0)	62 (12.4)	7 (5.4)	0.25 (0.11, 0.56) **
Residence, n (%)	Rural	417 (66.5)	310 (62.2)	107 (82.9)	2.95 (1.80, 4.83) ***
	Urban	210 (33.5)	188 (37.8)	22 (17.1)	1
Husbands’ education status, n (%)	No Formal Education	94 (15.0)	63 (12.7)	31 (24.0)	1
	Primary	235 (37.5)	170 (34.1)	65 (50.4)	0.77 (0.46, 1.3)
	Secondary	175 (27.9)	153 (30.7)	22 (17.1)	0.29 (0.16, 0.54) ***
	College or university	123 (19.6)	112 (22.5)	11 (8.5)	0.2 (0.09, 0.42) ***
Respondent’s Income, n (%)	Does not have her own source of income	270 (43.1)	205 (41.2)	65 (50.4)	1
	Has her own source of income	357 (56.9)	293 (58.8)	64 (49.6)	0.69 (0.45, 1.06)
Household main source of income, n (%)	Farming	342 (54.5)	249 (50.0)	93 (72.1)	1
	Employment	125 (19.9)	113 (22.7)	12 (9.3)	0.28 (0.15, 0.54) ***
	Small Business (Self-employment)	160 (25.5)	136 (27.3)	24 (18.6)	0.47 (0.28, 0.76) **
Ever used family planning, n (%)	No	254 (40.5)	215 (43.2)	39 (30.2)	1
	Yes	373 (59.5)	283 (56.8)	90 (69.8)	1.75 (1.16, 2.66) **
Family planning use at the time of conception, n (%)	No	393 (62.5)	330 (66.3)	63 (48.8)	1
	Yes	234 (37.3)	168 (33.7)	66 (51.2)	2.06 (1.39, 3.05) ***

CI, Confidence Interval, SD, Standard Deviation, OR, Odds Ratio

*P < 0.05

**P < 0.01

***P < 0.001.

### Univariate analysis

The numbers of women with and without unintended pregnancies are shown as counts and percentages in Table 1 with differences summarised using ORs. Among women with unintended pregnancies, 24% (31/129) had husbands with no formal education. The mean (standard deviation) age was 29.9 (5.2) years, only 5.4% (7/129) were college or university educated and 17.1% (22/129) resided in urban areas. Univariate analysis was carried out to identify independent variables that were associated with unintended pregnancy. Increasing age, rural residence, and use of family planning variables were associated with increased ORs (all P values < 0.01) for unintended pregnancy in unadjusted analyses (Table 1). Conversely, higher education among woman and their husbands, and higher household income were associated with decreased ORs (all P values < 0.01) for unintended pregnancy.

### Multivariate analysis

Having established that husbands’ education status was a strong predictor of unintended pregnancy, the next aim was to determine whether or not information on husbands’ education status was sufficient to identify high risk groups

A two-step process was followed to select variables for the final predictive model. First, starting with husbands’ education status, we checked which other variables maintained independent associations with unintended pregnancy. Including age of the respondent with husbands’ education status significantly improved prediction of unintended pregnancy (p < 0.001) as did residence (p = 0.025) and family planning use at conception period (p = 0.003) S1 Table. As age of the respondent appeared to be the strongest independent predictor, we retained it in the model. Next, for the model containing husbands’ education status and age of the respondent, we checked if the other two variables (residence and family planning use at the time of conception) were also independent predictors S2 Table. Each showed evidence of improving the model individually but adding both was not warranted. The three models that added to the relationship between husbands’ education status and unintended pregnancy are summarised in Table 2. For instance, the results showed that being married to a husband with at least some college or university education was associated with a decreased OR (0.36 [95%CI: 0.17, 0.82]) for unintended pregnancy after controlling for age and use of family planning at conception period (Model 3). Similarly, the results showed that being married to a husband with at least some college or university education was associated with a decreased OR (0.40 [95%CI: 0.18, 0.90]) for unintended pregnancy after controlling for age and rural residence (Model 3).

**Table 2:** Multivariable models.

Variables		Model 1	Model 2	Model 3
		Adjusted OR (95% CI)	Adjusted OR (95% CI)	Adjusted OR (95% CI)
Age		1.12 (1.07, 1.17) ***	1.11 (1.06, 1.16) ***	1.13 (1.09, 1.19) ***
Husbands’ education status	No Formal Education	1	1	1
	Primary	1.09 (0.63, 1.88)	1.08 (0.63, 1.88)	1.09 (0.63, 1.90)
	Secondary	0.60 (0.30, 1.18)	0.62 (0.31, 1.24)	0.66 (0.33, 1.33)
	College or university	0.36 (0.17, 0.81) *	0.36 (0.17, 0.82) *	0.40 (0.18, 0.90) *
Family planning use at conception period	No		1	
	Yes		1.56 (1.03, 2.36) *	
Residence	Rural			1.75 (0.98, 3.12)
	Urban			1
		-2 Log likelihood = 574.83, n = 627	-2 Log likelihood = 570.46, n = 627	-2 Log likelihood = 571.11 n = 627
		Pseudo R^2^ = 0.09	Pseudo R^2^ = 0.10	Pseudo R^2^ = 0.10

CI, Confidence Interval, OR, Odds Ratio

*P < 0.05

**P < 0.01

***P < 0.001.

Moreover, 28.2% (79/280) of rural vs 11.8% (11/93) of urban residents who reported ever use of family planning had an unintended pregnancy. Similarly, 37.8% (59/156) of rural vs 9.0% (7/78) of urban residents had an unintended pregnancy while using family planning methods.

## Discussion

This is the first study on the association of husbands’ education status with their wives unintended pregnancy in southern Ethiopia, to our knowledge. We found that the prevalence of unintended pregnancy was 20.6% in married women who were attending antenatal care. This rate of unintended pregnancy is higher than what had been reported for Northwest Ethiopia (13.7%) [25] and lower than for South Ethiopia (31.6%) and some parts of Oromia (41.5%, 27.9%, 27.1%, and 35%) [26–30]. Similarly, our observed unintended pregnancy rate in southern Ethiopia is higher than what has been reported in East Africa regions (11.2%) and among some developed regions (4.5%) of the world in 2010–14 [1]. The heterogeneity in unintended pregnancy rates could have resulted from differences in societal norms and cultural factors between countries.

We found that unintended pregnancy was most consistently associated with lowest husbands’ education status, confirming our study’s hypothesis. Being married to college or university educated men was associated with 60% decreased OR of having an unintended pregnancy after correcting for age of women and rural or urban residence. This could be explained by a positive correlation between being married to husband with a high education attainments and contraceptive use [31]. Similar findings have been shown in Jimma, Ethiopia where women having husbands who cannot read or write were 14 times more likely to have unintended pregnancy than their educated peers [32]. This finding is in contrast to previous research in high-income countries such as the United States where husbands’ education was not significantly associated with unintended pregnancy [33] suggesting that Ethiopia may have unique cultural norms and values that may increase the risk of unintended pregnancy. For instance, decisions about the use of contraceptives among women who did not use any family planning method are typically made by the husband according to the Ethiopia health and demographic survey [7]. Other research shows that Ethiopian women with poorly educated husbands, whom typically report having no autonomy, are three times more likely to have unintended pregnancies than those with highly educated husbands [26]. Conversely, women in high-income countries with supportive partners are more likely to use contraceptives effectively than those with unsupportive partners [34]. This suggests that women with least educated and unsupportive husbands may have specific family planning needs that are not currently being addressed.

Age of the respondent was strongly associated with unintended pregnancy over and above the effect of husbands’ education status. This is consistent with some studies conducted in Ethiopia where women older than 35 or 40 were two or four times more likely to have unintended pregnancy than younger aged women [25, 27]. Similarly, increasing age was also a predictor of unintended pregnancy in studies in Malawi [35] and Nepal [13]. This could be related to a decreased use of contraceptive with increasing age among women [36] or women aged 35 years and older could perceive themselves at a lower risk for a pregnancy [37]. By contrast, studies in Kenya [18], Brazil [38], and Canada [39] reported higher rates of unintended pregnancy in younger women. Whereas another study conducted in USA showed no association between age and unintended pregnancy [33]. These inconsistencies could also partially be explained by methodological differences between studies.

Both residence and family planning use around conception were marginally associated with unintended pregnancy after controlling for the effect of husbands’ education status and age of the respondent. This could be explained by poorer use of family planning services in rural vs urban areas in the study. Indeed, 79/280 (28.2%) of rural participants who ever used family planning had an unintended pregnancy compared to 11/93 (11.8%) of urban residents who ever used family planning. Moreover, 59/156 (37.8%) of rural residents had an unintended pregnancy while using family planning methods compared to only 7/78 (9%) of urban resident counterparts. This finding is consistent with previous research conducted in both northern [14] and southern Ethiopia [11].

There are interventions that targeted partners involvement which have shown positive outcomes such as mutual decision making on family planning [40]. Moreover, engaging men in family planning programmes can improve contraceptive practice, which might prevent unintended pregnancies [41, 42]. These findings suggest that interventions that involve couples may encourage male partners to better understand and the benefits of family planning, regardless of education status.

### Limitations

We acknowledge several study limitations and potential sources of bias. We analysed data from participants with complete information and used a cross sectional survey. Therefore, the true relationship between husbands’ education status and unintended pregnancy should be interpreted with caution. We also acknowledge that there might be residual confounding due to the influence of no paid employment (not collected in the survey) and close family members on the use of family planning. All data collections were by self-report and were collected from women who attended the health institution, which might not be representative of the general population.

## Conclusion

To our knowledge, our study is the first to show that unintended pregnancies were least likely to occur among women with husbands who had at least some college or university education in southern Ethiopia. We also found that increasing age and living in rural area were independently associated with unintended pregnancies. Strategies for addressing the family planning needs of families where husbands have minimal education should be the subject of future research. We believe that our findings provide useful information to policy makers in developing family planning interventions.

## Supporting information

S1 Table Analysis of deviance table showing the improvement of fit associated with adding other variables to husbands’ education status. (DOCX)

S2 Table Analysis of deviance table showing the improvement of fit associated with adding other variables to husbands’ education status and age of the respondent. (DOCX)

S1 Appendix Questionnaire. (DOCX)

S1 Dataset (SAV)

## List of abbreviations

ANC: Antenatal care
STROBE: Strengthening the Reporting of Observational studies in Epidemiology
ORs: Odds Ratios
