# Supplementary material for: Association of husbands' education status with unintended pregnancy in their wives in southern Ethiopia: A cross-sectional study
Source: PLoS One. 2020 Jul 9;15(7):e0235675. doi: 10.1371/journal.pone.0235675 (PMC7347164; doi:10.1371/journal.pone.0235675)
Supplement: S1 Table — (DOCX) [file pone.0235675.s001.docx]

The initial logistic regression model predicted unintended pregnancy status using husbands’ education status alone. Next each of the other potential predictors were added to the model singularly. The improvement in fit associated with adding the additional variable was tested using the likelihood ratio test. The addition of mothers age led to the greatest improvement in fit (decrease in deviance) and was clinically expected. So, respondents age was retained in the model.

**Table S1: Analysis of deviance table showing the improvement of fit associated with adding other variables to husbands’ education status**

|  |  | **Likelihood ratio test** | | |
| --- | --- | --- | --- | --- |
| **Models** | **Deviance** | **Change in deviance^*^** | **df** | **P value** |
| Husbands’ education status | 602.8 |  |  |  |
| + age | 574.8 | 27.9 | 1 | <0.001 |
| +Respondents education | 596.0 | 6.8 | 3 | 0.08 |
| +Residence | 597.8 | 5.0 | 1 | 0.03 |
| +Income | 599.9 | 2.9 | 1 | 0.09 |
| +Household main income | 600.0 | 2.8 | 2 | 0.25 |
| +Ever use Family planning | 601.6 | 1.2 | 1 | 0.3 |
| +Family planning at conception period | 594.2 | 8.6 | 1 | 0.003 |

^*^ Follows the chi-square distribution
