# Supplementary material for: Association of husbands' education status with unintended pregnancy in their wives in southern Ethiopia: A cross-sectional study
Source: PLoS One. 2020 Jul 9;15(7):e0235675. doi: 10.1371/journal.pone.0235675 (PMC7347164; doi:10.1371/journal.pone.0235675)
Supplement: S2 Table — (DOCX) [file pone.0235675.s002.docx]

**Table S2: Analysis of deviance table showing the improvement of fit associated with adding other variables to husbands’ education status and age of mother.**

|  |  |  | **Likelihood ratio test** | |
| --- | --- | --- | --- | --- |
| **Models** | **Deviance** | **Change in deviance** | **df** | **P value** |
| Husbands’ education status + age | 574.8 |  |  |  |
| +Residence | 571.1 | 3.7 | 1 | 0.05 |
| +Family planning at conception period | 570.5 | 4.4 | 1 | 0.04 |
