# Supplementary material for: Association of husbands' education status with unintended pregnancy in their wives in southern Ethiopia: A cross-sectional study
Source: PLoS One. 2020 Jul 9;15(7):e0235675. doi: 10.1371/journal.pone.0235675 (PMC7347164; doi:10.1371/journal.pone.0235675)
Supplement: S1 Appendix — (DOCX) [file pone.0235675.s003.docx]

## Questionnaire

| SN | **Section 1: Background information of Pregnant Women** | **Responses** | | Skip |
| --- | --- | --- | --- | --- |
| 101 | Age in years completed | | ___________ |  |
| 103 | Have you ever attended formal school? | | 1. No . . . . . . . . . . 2. Yes | Skip to 104 |
| 103 | If Yes, what is the highest level of school you attended? | | 1. Primary 2. Secondary 3. College and university |  |
| 104 | What is your current marital status? | | 1. Single 2. Married 3. Divorced/Separated 4. Widowed |  |
| 107 | Place of residence | | 1. Urban 2. Rural |  |
| 109 | Main source of Income of the household | | 1. Faming 2. Employment 3. Business (Self-employment) |  |
| 110 | Do you have your own source of income? | | 1. Yes 2. No |  |
| 112 | Educational status of respondent’s husband | | 1. No formal education 2. Primary 3. Secondary 4. College and university |  |
| 309 | Have you ever used a planning method? | | 1. Yes 2. No |  |
| 310 | Have you been using any family planning method when the pregnancy occurred? | | 1. Yes 2. No |  |
| 311 | Did you want to be pregnant? | | 1. Yes 2. No |  |

**መጠይቅ**

| ተ.ቁ | ክፍል 1፡ የነፍሰጡር እናቶች ጠቅላላ መረጃ | ምላሽ | ወደ |
| --- | --- | --- | --- |
| 101 | ዕድሜዎ ምን ያህል ነው? | ______________________ |  |
| 103 | ትምህርት ተከታትለው ያውቃሉ? | 1. አይደለም. . . . . . . . . . . . . . . 2. አዎ | ወደ 104 ይሂዱ |
| 103 | መልስዎ አዎን ከሆነ የትምህርት ደረጃዎ ምንድነው? | 1. የመጀመሪያ ደረጃ 2. ሁለተኛ ደረጃ 3. ኮሌጅ እና ከዛ በላይ |  |
| 104 | የጋብቻ ሁኔታዎ | 1. ያላገባች 2. ያገባች 3. የፈታች/የተለያየች 4. የትዳር አጋሯን በሞት ያጣች |  |
| 107 | የመኖሪያዎ ስፍራ | 1. ከተማ 2. የገጠር መንደር |  |
| 109 | ዋናው የገቢ ምንጭ ምንድ ነው | 1. ግብርና 2. ቅጥር 3. ንግድ (የግል ስራ) ______ |  |
| 110 | የራስዎ የገቢ ምንጭ አለዎት? | 1. አይደለም 2. አዎ |  |
| 112 | የባለቤትዎ ትምህርት ደረጃ | 1. መጻፍ እና ማንበብ የማይችል 2. የመጀመሪያ ደረጃ 3. ሁለተኛ ደረጃ 4. ኮሌጅ እና ከዛ በላይ |  |
| 309 | የወሊድ መቆጣጠሪያ ተጠቅመው ያውቃሉ? | 1. አይደለም 2. አዎ |  |
| 310 | እርግዝናው ሲፈጠር የወሊድ መቆጣጠሪያ እየተጠቀሙ ነበር? | 1. አይደለም 2. አዎ |  |
| 311 | ይህንን እርግዝና ዓቅደውት ነበር? | 1. አይደለም 2. አዎ |  |
